# Heating Control Strategy Based on Dynamic Programming for Building Energy Saving and Emission Reduction

**DOI:** 10.3390/ijerph192114137

**Published:** 2022-10-29

**Authors:** Haosen Qin, Zhen Yu, Tailu Li, Xueliang Liu, Li Li

**Affiliations:** 1 Tianjin Key Laboratory of Clean Energy and Pollutant Control, School of Energy and Environmental Engineering, Hebei University of Technology, Tianjin 400301, China; 2 Institute of Building Environment and Energy, China Academy of Building Research, Beijing 100013, China

**Keywords:** HVAC system, nearly zero energy building, competitive learning, dynamic programming, model predictive control, simulation

## Abstract

Finding the optimal balance between end-user’s comfort, lifestyle preferences and the cost of the heating, ventilation and air conditioning (HVAC) system, which requires intelligent decision making and control. This paper proposes a heating control method for HVAC based on dynamic programming. The method first selects the most suitable modeling approach for the controlled building among three machine learning modeling techniques by means of statistical performance metrics, after which the control of the HVAC system is described as a constrained optimization problem, and the action of the controller is given by solving the optimization problem through dynamic programming. In this paper, the variable ‘thermal energy storage in building’ is introduced to solve the problem that dynamic programming is difficult to obtain the historical state of the building due to the requirement of no aftereffect, while the room temperature and the remaining start hours of the Primary Air Unit are selected to describe the system state through theoretical analysis and trial and error. The results of the TRNSYS/Python co-simulation show that the proposed method can maintain better indoor thermal environment with less energy consumption compared to carefully reviewed expert rules. Compared with expert rule set ‘baseline-20 °C’, which keeps the room temperature at the minimum comfort level, the proposed control algorithm can save energy and reduce emissions by 35.1% with acceptable comfort violation.

## 1. Introduction

With the gradual extension of people’s living and working hours in buildings, the requirements on the healthier and better thermal comfort indoor environment lead to increasing energy consumptions of the building sector. According to the forecast of the International Energy Agency [1], the world energy structure is undergoing tremendous changes, and a new form of global energy is emerging. The new form of energy described in the Net Zero Emissions by 2050 Scenario (NZE) is a cooperative economy in which countries work together to achieve emissions reductions where necessary. One important measure to achieve emissions reduction is to focus on energy efficiency and adjust the energy service demand through behavioral change [2]. The building sector is responsible for 33% of global CO2 emissions, and HVAC systems are the crucial energy consumer in buildings with the largest share [3]. Nearly zero energy buildings (NZEB) offer a significant opportunity for both energy use and emissions reduction [4]. NZEBs have high performance envelopes where the thermal storage capacity of the building itself and its facilities (i.e., floors, ceilings, walls and furniture) can be fully utilized, providing the potential to improve the overall control performance of its HVAC systems. This energy-saving potential of HVAC systems can be tapped through smart control [5]. A model-based method, such as model predictive control (MPC), is an online optimization method whose basic idea is to use the model of the system under study to predict the future state and minimize a certain cost function within the forecast period. This method is a real-time rolling optimization process, and its optimization results depend on the prediction accuracy of the model used.

### 1.1. Building Energy Simulation and Modeling

The thermal response model of the building is crucial for the intelligent control of the HVAC system. The thermal response model provides key indicators, such as the energy demand and temperature of buildings. Simulation tools are used to provide data for the development of energy and environment models of buildings. The widely used building energy simulation tools include TRNSYS [6], EnergyPlus [7] and IES<VE> [8]. A comparison of the simulation tools can be found in [9]. Most of the building and equipment models used by simulation tools are first principle models, which require correct description of the component following physical laws and precise input parameters under the descriptive structure. However, the detailed and precise information of the specific equipment at certain working conditions are hard to acquire.

On the other hand, data-driven methods do not require precise physical information of the building and equipment. They mainly rely on the regression and summarization of historical data to predict required information [10,11,12]. Two commonly used data-driven methods for the discussed topic are statistical methods and supervised machine learning method [13,14,15]. Although statistical methods are relatively easy to implement, they are more difficult to handle complex nonlinear relationships, while the thermal behavior of buildings is usually complex and nonlinear. The most widely used data-driven models include support vector machine (SVM), artificial neural network (ANN) and multivariate polynomial regression (MPR) [16,17,18,19,20].

Each building has a unique load due to differences in function, location and economic conditions. A modeling technique that performs well in one building may not perform as well in other buildings. Until a comprehensive, integrated and generally accepted data set is available, it is necessary to select modeling techniques independently for different buildings.

### 1.2. Dynamic Programming Applied to HVAC

MPC describes the control of an HVAC system as a constrained optimization problem in which the future state is predicted by a model of the system under study, and the controller action is given by solving the optimization problem. Models of building and HVAC equipment usually involve physical principles of heat transfer, thermodynamics and fluid dynamics including nonlinearity and nonconvexity, so the biggest challenge for developing controllers based on optimization (e.g., MPC) is the solution of nonconvex problems. The common solutions to optimization problems are traditional mathematical methods (e.g., Newton–Raphson method [21] and interior-point method [22]) and heuristic algorithms (e.g., genetic algorithms [23], ant colony algorithms [24], particle swarm algorithms [25], etc.). Mathematical methods are logical and precise models, but the required objective function expressions are difficult to abstract in many optimization scenarios and cannot be applied effectively. Heuristic methods have good performance and are applicable to most optimization problems, but usually the solutions of optimization problems obtained by these methods are locally optimal rather than globally optimal, and the robustness of these methods is poor due to the lack of rigorous mathematical proofs. Except for nonconvexity, the optimization problem of HVAC system must dynamically consider the interrelationship between the before and after decisions. For example, the energy consumption of an HVAC system depends on the current temperature setting of the room and the previous thermal storage state of the room. The dynamic programming algorithm is based on Bellman’s optimization principle, which decomposes the optimization problem into a number of interrelated subproblems and solves the subproblems iteratively to obtain the solution of the original optimization problem [26]. The dynamic programming algorithm is suitable for solving such multistep decision, nonconvex problems. The stability and robustness of dynamic programming algorithm have been demonstrated in related works [27].

In recent years, researchers have conducted studies on the application of dynamic programming algorithms to the HVAC system. Chen et al. established an optimization analysis method for ice thermal storage air conditioning system that optimize the performance of the ice storage tank and the life cycle cost using dynamic programming approach [28]. The optimal chiller and ice storage tank capacity were obtained from the simulation results. Pombeiro et al. used dynamic programming with simplified thermal model and genetic algorithm with EnergyPlus to optimize the control of the HVAC system. The optimization performance of the two algorithms were compared [29]. Since the simplified thermodynamic model used by the dynamic programming algorithm cannot reflect the thermal inertia of the building, the optimization effect of the dynamic programming algorithm is not as good as the genetic algorithm.

Although some progress has been made in the application of dynamic programming algorithms, there are still issues that need to be addressed. First, the dynamic programming algorithm has difficulties in obtaining the historical state of the building due to the no aftereffect requirement. Most of the existing research introduce some simplifications or some unrealistic assumptions during the controller design, which limit the generality of the proposed approach. For example, De Ridder et al. [30] used a very simple first-order model to predict the state of the system. However, the simplified model has difficulty in reflecting the thermal inertia, which has a very negative impact on the final optimization. Second, the control objects of the existing dynamic programming controllers are mostly limited to air conditioning equipment and have not explored the potentials of operation of the buildings. To further investigate these two aspects, and hence facilitate the use of MPC in buildings, this research herein presents a model predictive controller using dynamic programming based on data-driven models.

### 1.3. Research Gap and Objective

This paper uses a nearly zero energy residential building as the case to demonstrate applicability and benefits of using dynamic programming in buildings. The building is modeled using TRNSYS, a transient system energy modeling software designed to solve complex energy system problems [31]. Detailed verifications of TRNSYS can be found in [32,33]. The output data of TRNSYS are used to generate MLR, SVR and ANN models for building temperature and HVAC system energy consumption. The four indicators of Mean Square Error (*MSE*), Mean Absolute Error (*MAE*), Mean Absolute Percent Error (*MAPE*) and Coefficient of Determination (*R*^2^) are used to evaluate and select the most suitable model for the MPC controller. On this basis, using the indoor state as input, a multistep predictive optimization control method for nearly zero energy residential buildings is established based on a dynamic programming algorithm.

The goal of this paper is to propose an automated controller that can combine building thermodynamics with HVAC systems. It automatically finds optimal working mode of HVAC system and make use of the natural cooling and natural heating. The controller explores the energy-saving potential of HVAC systems without sacrificing the required thermal comfort and IAQ. To this end, this paper attempts to explore further in the following areas:

(1) Three model identification techniques are implemented in parallel, with algorithms for automatic training and selection of potential models to ensure the prediction accuracy of the models on different data sets.
(2) The introduction of ‘thermal energy storage in building‘ solves the problem that, using dynamic programming, it is difficult to obtain the historical state of the building due to there being no aftereffect requirement.
(3) Problem formulation, including the definitions of decision, state, stage, state transition rules and cost function for dynamic programming, is carefully designed to ensure that the controller can adapt to the complex thermal dynamics and environmental uncertainties involved in HVAC control.
(4) With a case study of a nearly zero energy building, a systematic analysis of the controller’s performance in terms of thermal comfort and energy consumption is made to demonstrate the applicability and benefits of dynamic programming in buildings.

## 2. Basics of Three Data-Driven Models

### 2.1. Multivariate Linear Regression

The multiple linear regression method is a modeling technique used to describe the influence of variables as independent data on the prediction target of the model using linear representations. This method maintains the fast performance of linear methods while allowing them to fit a wider range of data. The data of HVAC energy consumption and building thermal behavior are not strictly of a linear relationship. The approach is to use the nonlinear function (basis function) of each dimension feature as a secondary variable, and then perform linear regression analysis on the updated variable set.

By using the linear fitting in the high-dimensional space constructed by these basis functions, the model can flexibly fit a wider range of data. After the model form is decided, the coefficients can be solved by the least square method.

### 2.2. Support Vector Regression

The support vector machine technique was originally developed by Vapnik et al. [34], which is one of the most widely used machine learning techniques for classification, estimation and nonlinear regression problems. The basic idea of support vector machine is to transform the input space into high-dimensional feature space through transformation and extract the information and regularity contained in the data [35].

Given a data set with $\bar{N}$ elements $\left\{(x_{i},y_{i})\;\;\;\;i=1,2,\ldots ,\bar{N}\right\}$, *N* represents the number of samples in the training data set, and *x_i_* is the *i*-th element in the *N*-dimensional vector. SVM approximates the regression function by mapping the training data *x_i_* into a high one-dimensional feature space. The feature space forms an optimized hyperplane that characterizes the nonlinear relationship between the input (independent variable) and the output data (dependent variable):

(1) f(x)=ωϕ(x)+b

where *f*(*x*) represents the forecasting values, *x* is the input parameter, *ϕ*(*x*) is a mapping function that maps *x* to a high one-dimensional feature space, *ω* is the weight coefficient and *b* is the adjustable factor.

The ε insensitive loss function is defined as Equation (6).

(2) $$L_{\varepsilon }(y_{i},f(x_{i}))=\max(0,|y-f(x)|-\varepsilon )$$

The values of *ω* and *b* can be estimated by minimizing the regularized risk function:

(3) $$\frac{1}{2}||\omega |{|}^{2}+C\frac{1}{n}\sum_{i=1}^{n}L_{\varepsilon }(y_{i},f(x_{i}))$$

where ${\left||\omega |\right|}^{2}$ represents the regularized term. Minimizing the value of this term can make the function as flat as possible. *C* represents the regularization constant, and ɛ is the threshold of the support vector machine. The term $\frac{1}{n}\sum_{i=1}^{n}L_{\varepsilon }(y_{i},f(x_{i}))$ is the empirical error of the insensitive loss function measurement.

By introducing a positive slack variable ζ_i_* and Langrangian multipliers, such as α_i_ and α_i_*, the SVR regression equation can finally be written as Equation (4) [36]:

(4) $$f(x)=\sum_{i=1}^{n}(\alpha_{i}-\alpha_{i}^{*})K(x_{i},x)+b$$

where $K(x_{i},x)$ is called the kernel function, which can nonlinearly map the training data to a high-dimensional space. Radial Basis Functions (RBFs, in our case, Gaussian), as well as Polynomial, Linear and Sigmoid functions, are generally used as kernels. The choice of kernel function should be selected according to its evaluation criteria [37].

### 2.3. Artificial Neural Networks

Artificial Neural Networks (ANN) are universal approximators. Multi-layer-perceptons (MLPs) are a widely used ANN form for MPC [38]. MLPs can approximate nonlinear static mapping between input and output variables.

The training of ANN usually needs to go through two stages. The first stage is the forward propagation of the signal, from the input layer to the hidden layer, and finally to the output layer. The inputs from the previous layer are multiplied by the weights, summed up and added with a bias. The calculation result of the previous layer is passed to the next layer through the activation function in the hidden layer. In general, vector X is composed of M input variables and N neurons, and vector Y is composed of L output variables. The calculation formula of the neural network is:

(5) $$y=\sigma (xw^{T}+b_{0})\theta +b_{1}\;\;\;\;\;\;\;s.t.\;\;\;\;w\in R^{N\times M},b_{0}\in R^{1\times N},\theta \in R^{N\times 1},b_{1}\in R^{1\times 1}$$

Here, *σ*() is the activation function; *ω*, *b*_0_, *θ* and *b*_1_ are variables of the neural network itself. Neural network training means to “fit” these variables with the training data.

The second stage is the back propagation of errors, from the output layer to the hidden layer, and finally to the input layer. The weights and biases from the hidden layer to the output layer and the weights and biases from the input layer to the hidden layer are adjusted in turn. Common training algorithms include the back-propagation algorithm [39], Levenberg–Marquardt (LM) algorithm [34,40], Bayesian regularization algorithm [22,41] and Broyden–Fletcher–Goldfarb–Shanno (BFGS) algorithm [25]. According to the above algorithm, a large amount of data can be used to solve the iterative design process; these data are divided into several groups, some of which are used for training, and some are used to verify the solution. This research uses the back-propagation algorithm to train the ANN model. The back-propagation algorithm has two elements, in simple terms: the error function is calculated in the forward direction, and the gradient descent is derived in the reverse direction [39].

### 2.4. Evaluation Criteria

Four accuracy metrics are used to evaluate the performance of three data-driven models [19]. The first is the predictive coefficient of determination (*R*^2^), which indicates how close the predicted value is to the actual value. The larger the *R*^2^, the better the model. *R*^2^ can be calculated according to Equation (6):

(6) $$R^{2}=\frac{\sum_{i=1}^{n}{\left[y_{\mathrm{predicted}}-{\bar{y}}_{\mathrm{observed}}\right]}^{2}}{\sum_{i=1}^{n}{\left[y_{\mathrm{observed}}-{\bar{y}}_{\mathrm{observed}}\right]}^{2}}\;\;\;\;\;i=1,2,\ldots ,n$$

where $y_{\mathrm{observed}}$ is the observed values, ${\bar{y}}_{\mathrm{observed}}$ is the average of the observed values, $y_{\mathrm{predicted}}$ is the predicted values, and n stands for the total number of observations.

The second evaluation index is the Mean Square Error (*MSE*), which is used to evaluate the error between the observed value and the predicted value. The smaller the *MSE*, the better the model. *MSE* can be described by Equation (7):

(7) $$MSE=\frac{\sum_{i=1}^{n}{\left[y_{\mathrm{observed}}-y_{\mathrm{predicted}}\right]}^{2}}{n},\;\;\in \;[0,+\infty ),\;\;\;\;\;\;i=1,2,\ldots ,n$$

The last two accuracy indicators are Mean Absolute Error (*MAE*) and Mean Absolute Percent Error (*MAPE*). *MAE* is used to measure the average absolute error between the predicted value and the true value. The smaller the *MAE*, the better the model. Its definition is as follows:

(8) $$MAE=\frac{\sum_{i=1}^{n}\left||y_{\mathrm{observed}}-y_{\mathrm{predicted}}|\right|}{n},\;\;\;\;\;\;\;\;\in [0,+\infty ),\;\;\;\;\;\;i=1,2,\ldots ,n$$

Mean Absolute Percent Error (*MAPE*) is similar to *MAE*, except that it is standardized on the basis of *MAE*. The percentage error has the advantage of being independent of the scale, so it is often used to compare the performance between different models. The definition of *MAPE* is as follows:

(9) $$MAPE=\frac{100\%}{n}\sum_{i=1}^{n}\left\|\frac{y_{\mathrm{observed}}-y_{\mathrm{predicted}}}{y_{\mathrm{observed}}}\right\|\;\;\;\;,\;\;\;\;\in [o,+\infty )\;\;\;,\;\;\;i=1,2,\ldots ,n$$

The best model is a model that collects the minimum values of error metrics (i.e., *MSE*, *MAE*, *MAPE*) and the maximum value of predictive R^2^.

## 3. Methodology

### 3.1. Model Validation

In order to demonstrate the applicability of the proposed discrete-time dynamic programming algorithm to the indoor climate control, this research tests the idea on a nearly zero energy residential building using TRNSYS as the simulation environment.

#### 3.1.1. Building and TRNSYS model

The simulated residential building is a part of the Future Building Laboratory (FBL) of the Chinese Academy of Building Research (CABR) in Beijing, China. The FBL is a full-scale experiment facility used for the study of the innovative energy and environment technologies in residential buildings. The FBL includes six similar apartments with different envelope and energy systems.

**Figure 1:**
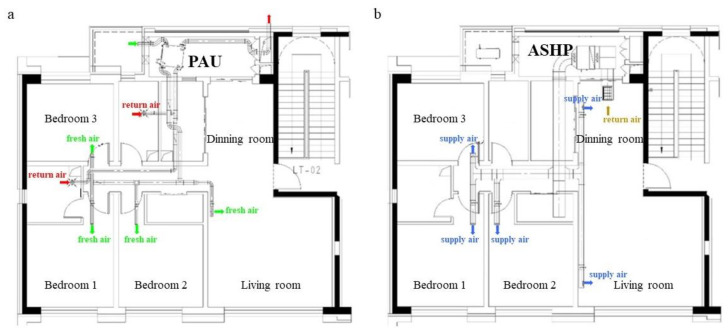
HVAC system diagram.

This study selects two apartments as the simulation targets. The apartments use an Air-Sourced Heat Pump (ASHP) to provide heating air using duct and Primary Air Unit (PAU) with heat recovery, as shown in Figure 1.

**Figure 2:**
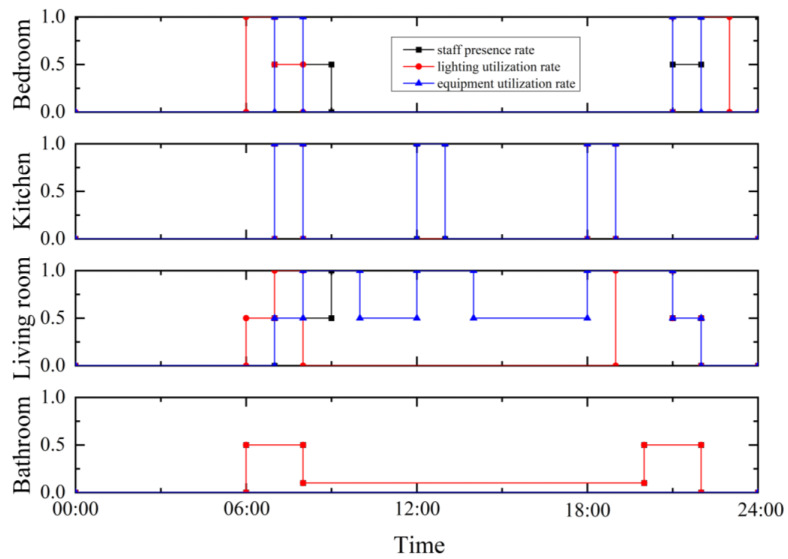
Occupation, lighting and equipment schedule.

Each apartment includes three bedrooms, a living room, a dining room, a kitchen and two bathrooms. The main parameters of building envelope, ASHP and PAU are given in Table 1. The occupation rate, lighting utilization rate and equipment utilization rate of each room are set in accordance with the “Design Standard for Energy Conservation of Residential Buildings in Severe Cold and Cold Areas” JGJ26-2018 standard, as shown in Figure 2. The lighting density value of each room is set according to the target value of “Architectural Lighting Design Standard” GB 50034-2013 standard. The power density of each room equipment is set according to the function of the room, and the heat dissipation of the human body is set in accordance with ISO7730. For the specific settings, see Table 2.

**Figure 3:**
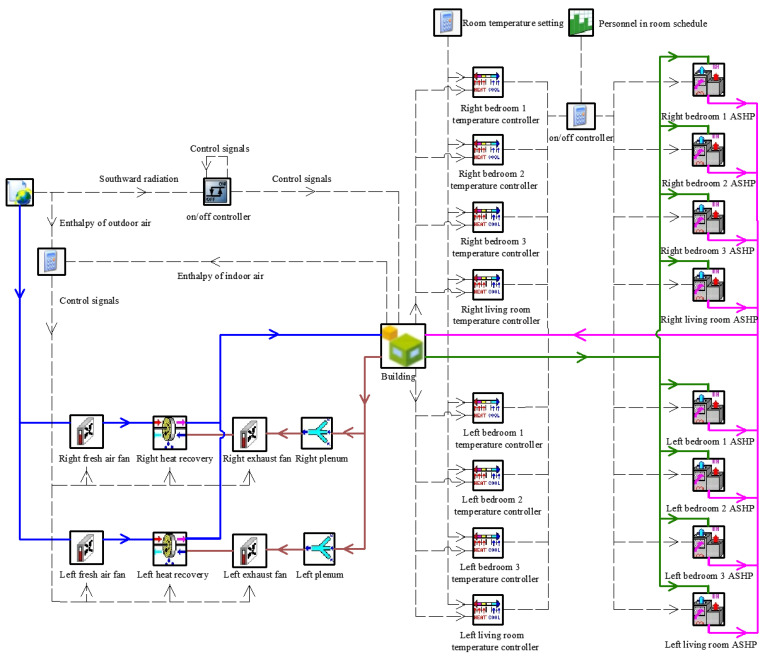
HVAC system model in TRNSYS.

The HVAC system of the selected apartments in TRNSYS environment are given in Figure 3. The HVAC system of the selected apartment consists of three main subsystems: air conditioning system, fresh air system and shading system. The air conditioning system consists of a five-stage thermostat controller (Type 108), an air-source heat pump model (Type 954), a start–stop controller and a personnel-in-room schedule, which turns on the AHSP when the room is occupied and the temperature meets the temperature control requirements. The fresh air system consists of air mixing valve (Type 648), variable air volume fan (Type 147), heat recovery unit (Type 667b) and controller, which controls the start–stop function of the fan and heat recovery unit according to the enthalpy difference between indoor and outdoor air (the fresh air system is in heat recovery mode when all equipment is on, and in bypass mode when only the fan is on). Shading control is achieved by an on/off differential controller (Type2). The differential controller allows for setting a certain value interval when the outdoor solar radiation is higher than this interval to turn on the shading and lower than this interval to turn off the shading; otherwise, it does not change the device status.

**Figure 4:**
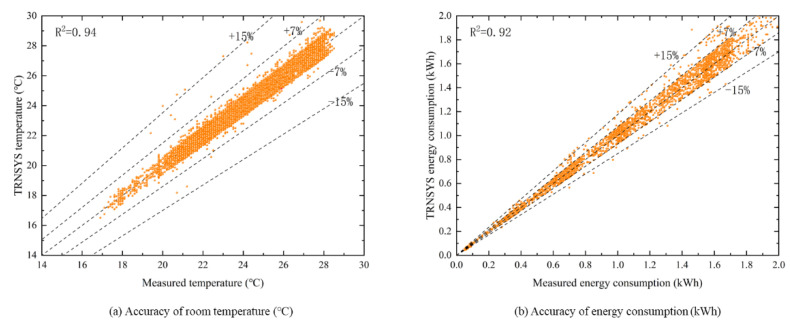
Accuracy validation of TRNSYS model.

The TRNSYS simulation model was validated using historical operational data of the building in 2021, as shown in Figure 4. Figure 4a shows the TRNSYS simulated value of room temperature compared with the measured value (taking the master bedroom of the right household as an example), and Figure 4b shows the TRNSYS simulated value of HVAC system energy consumption compared with the measured value.

#### 3.1.2. Simulation Date

A whole year simulation is carried out. In parameter identification, the system inputs include: (1) outdoor weather conditions, including outdoor air temperature, outdoor air enthalpy, outdoor air relative humidity, solar horizontal radiation and southward solar radiation; (2) internal gain ($S_{k}^{In,gain}$), including lighting load, equipment load and human body heat dissipation; (3) the heating and cooling output of the air conditioning equipment (*Q*_k_), which is calculated from the volume of the circulated air flow and the temperature difference between the supply and return air. The system outputs include: (1) room air temperature of the main zone and (2) the energy consumption of air conditioning equipment. 

The data generated by the TRNSYS simulation program that cover most of the state-actions conditions is used to train the prediction model, as shown in Table 3.

#### 3.1.3. Model Regression

The three data-driven methods used in this research are implemented in the Python 3.8 environment with the help of Scikit-learn library. The setting of hyperparameters has a great influence on the accuracy of the model and training efficiency. In this research, an automatic adjustment mechanism of hyperparameters was established to reduce the dependence of the know-hows to find optimal hyperparameters. For the MLR model, regression models with the highest degree of 1–5 are tested, respectively, and the one with the highest precision is selected by the algorithm. For the ANN model, this paper uses an algorithm called Best Network after Multiple Iterations (BNMI) to determine the optimal parameters of the ANN (number of hidden layers, number of nodes, node interconnection weights and bias) [42]. For the SVR model, this paper uses a grid search algorithm to automatically adjust the parameters [43].

The thermal energy storage in building $T_{k}^{his}$ is used to characterize the historical state of the building. $T_{k}^{his}$ is determined by the average temperature at 4 h before each optimization cycle; see Table 4. The input variables of the model are shown in Table 5.

The structure and accuracy indicators of different models are shown in Table 6. The ANN model has the best accuracy. The coupling of the room temperature model with the equipment energy consumption model is divided into two cases: (1) When the ASHP is off, the temperature model directly calculates the room temperature at the next time step, and the equipment energy consumption is zero. (2) When the ASHP is on, the temperature model calculates the maximum temperature (ASHP running at maximum power, PAU off, shading off) and the minimum temperature (ASHP off, PAU on, shading on) that the room can reach at the next time step. If the set temperature is in the possible temperature range or above the maximum temperature, the energy consumption is calculated by the energy consumption model according to the equipment state setting; otherwise, the equipment energy consumption is zero.

**Figure 5:**
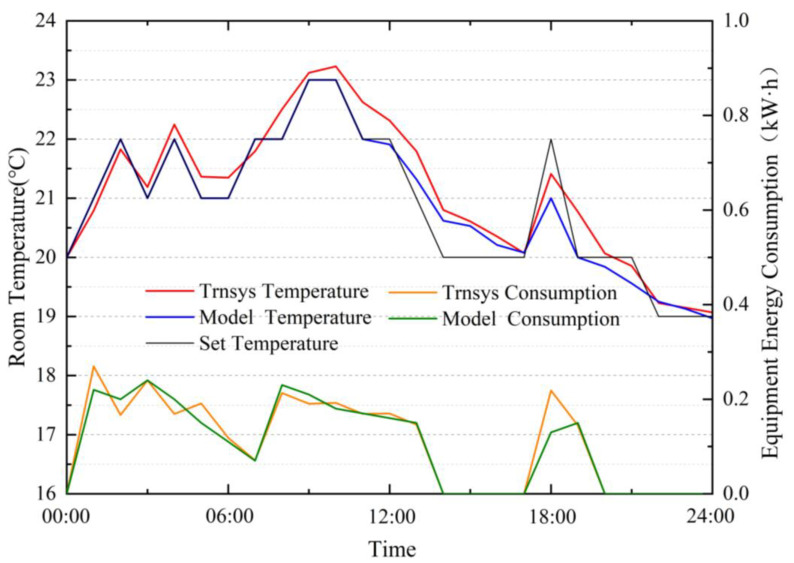
Comparison of simulation results between TRNSYS simulation and identified models.

In order to verify the models, this study used the same settings in the TRNSYS simulation platform and simulated both the models provided by the TRNSYS and the identified models on the same day. The results are shown in Figure 5. The identified models have good accuracy and can support the development of subsequent control algorithms.

### 3.2. Control Method

The purpose of this research is to find the best balance between user’s comfort, lifestyle preferences and energy costs of HVAC systems. This problem can be regarded as a complex multistage decision problem, in which the interrelationship of decisions obtained by previous and subsequent iteration steps must be considered dynamically. For example, the energy consumption of the HVAC system is dependent on the room temperature setting and the previous room heat storage status. For this multistage decision problem, discrete-time dynamic programming is a suitable framework.

#### 3.2.1. Dynamic Programming Method

Based on Bellman’s principle of optimality [25], dynamic programming (DP) decomposes the original problem into several interrelated subproblems and obtains the solution of the original multistage decision-making problem by solving the sub-problems iteratively. Dynamic programming is particularly well suited for situations where decisions need to be made in stages. The outcome of each decision may not be completely predictable, but it can be anticipated to some extent before the next decision is made. The objective is to minimize a certain cost. DP algorithm considerably reduces the complexity of multistage decision problems. In discrete-time dynamic programming, there are five main concepts: decisions, states, stages, state transition and strategies.

A DP problem involves a discrete-time dynamical system of the form:

(10) $$x_{k+1}=f_{k}(x_{k},u_{k}),k=0,1\ldots ,N-1$$

where k represents the stage index; *x*_k_ represents the state of the system; *u*_k_ represents the decision variable, selected at time k from some given set *U*_k_(*x*_k_) that depends on *x*_k_; *f*_k_ represents a function of (*x*_k_, *u*_k_), describing the mechanism by which the state is updated from time k to time k+1; *N* represents the number of times the control is applied.

This problem also involves an additive cost function, that is, the cost incurred at stage k, denoted by *g*_k_(*x*_k_*, u*_k_), accumulates over time:

(11) $$J(x_{0};u_{0},\ldots ,u_{N-1})=g_{N}(x_{N})+\sum_{K=0}^{N-1}g_{K}(x_{k},u_{k})$$

**Figure 6:**
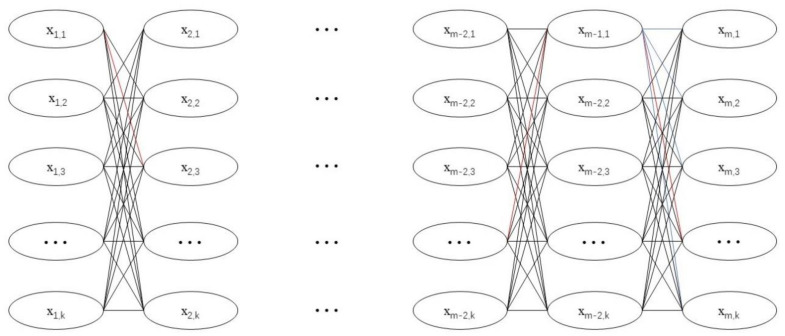
Finding optimal control strategy using DP algorithm.

Figure 6 shows the description of DP for the specific problem discussed in this paper. *X* represents the combination of the indoor temperature and the remaining fresh air opening time. The first subscript represents the time, and m is the length of the time series. The second subscript of each state number is the number of states, assuming there are k states. In this research, one day is divided into 24 stages with time step of one hour, m = 24.

DP obtains the optimal value by minimizing the cost over all sequences {*u*_0_,…, *u*_N−1_} that satisfy the control constraints:

(12) $$J_{k}^{*}(x_{k})\;=\underset{u_{k}\in U_{k}(x_{k})}{\min}\left[g_{k}(x_{k}^{},u_{k})+J_{}^{*}(x_{k+1};u_{k+1},\ldots ,u_{N-1})\right],\;for\;all\;x_{k}$$

where ‘*’ represents the optimal solution of all states and their decisions in the k^th^ stage, and $J_{k}^{*}$ represents the lowest cost from stage k to stage *m*. Note that at stage k, the calculation in Equation (12) must be done for all states *x*_k_ before proceeding to stage k−1.

Once the function $\left\{J_{0}^{*},\ldots ,J_{N}^{*}\right\}$ has been obtained, the algorithm can construct the optimal control sequence $\left\{u_{0}^{*},\ldots ,u_{m-1}^{*}\right\}$ and the corresponding state trajectory $\left\{x_{1}^{*},\ldots ,x_{m}^{*}\right\}$ for the given initial state *x_0_* in the following way:

(13) $$u_{i}^{*}\in \arg\;\underset{u\in U(x_{i}^{*})}{\min}\left[g_{i\;\mathrm{to}\;i+1}^{*}+J_{i+1}^{*}(x_{i+1})\right]$$

#### 3.2.2. DP-Based Description Control Model

When the dimensionality of the state variables increases, the computational complexity of dynamic programming problems increases exponentially. Therefore, the selection of state variables should be as streamlined as possible. The state in this study is defined as the current temperature of the room ($S_{k}^{in,T})$ and the remaining start hours of the PAU ($S_{k}^{PAU,times})$ in the simulated day because of the following: (1) $S_{k}^{in,T}$ is not only an essential variable affecting the operation of HVAC system, but also an essential variable reflecting indoor comfort; (2) converting the minimum fresh air volume into the minimum fresh air opening hours can reduce the computational cost of the algorithm and meet the constraints.

(14) $$x_{k}={\left[S_{k}^{in,T},S_{k}^{PAU,times}\right]}^{T}$$

The decision variable is the mode of devices in the system, including the on/off function of the ASHP, the setting of the room temperature, the on/off function of the PAU, the working mode of the PAU (bypass or heat recovery) and percentage of solar radiation blocked by shading equipment (0%, 10%, 20%, …, 100%).

(15) $$U_{k}={\left[S_{k}^{ASHP,on/off},S_{k}^{Set,T},S_{k}^{PAU,on/off},S_{k}^{PAU,mode},S_{k}^{Shading}\right]}^{T}$$

From time k to time k+1, the state transition must meet the constraints. Firstly, the room temperature should be higher than the lower limit temperature when the ASHP is off and the PAU is in bypass mode. The room temperature should be lower than the higher limit temperature when the ASHP is on and PAU is off. The specific value can be calculated by the previously established room temperature model. Secondly, the remaining fresh air opening time should be equal to or one less than the previous stage.

(16) $$\left\{\begin{array}{l} S_{{}_{k+1,\min}}^{in,T}\leq S_{{}_{k+1}}^{in,T}\leq S_{{}_{k+1,\max}}^{in,T}\\ S_{k+1}^{PAU,times}=S_{k}^{PAU,times}\;\;\;or\;\;\;S_{k+1}^{PAU,times}=S_{k}^{PAU,times}-1 \end{array}\right.$$

The algorithm minimizes cost function by finding the optimal state trajectory given the 24 h weather forecast information. The algorithm calculates the discomfort penalty from the temperature model and the energy cost from the energy consumption model.

(17) $$g_{k}(x_{k},u_{k},S_{k}^{Weather})=\lambda_{}^{cmf}C_{k}^{cmf}+\lambda_{}^{cst}C_{k}^{cst}+\lambda_{}^{on/off}C_{k}^{on/off}$$

where, *λ^cmf^*, *λ^cst^* and *λ^on/off^* respectively represent the weighting coefficient of the discomfort cost, energy cost and ASHP on/off cost. One of the purposes of setting the weighting coefficient is to keep the three cost terms in the same order of magnitude, so *λ^cmf^* is set in the range 1–10, *λ^cst^* is set in the range 50–150 and *λ^on/off^* is generally set to 10. Another purpose is for adjusting the user’s usage preferences between energy efficiency and thermal comfort. Functions $C_{k}^{cmf}$, $C_{k}^{cst}$ and $C_{k}^{on/off}$ respectively represent the discomfort cost, energy cost and ASHP on/off cost of stage k. $C_{k}^{cst}$ can be calculated by the energy consumption model proposed in the previous chapter. $C_{k}^{cmf}$ and $C_{k}^{on/off}$ are given by the following Equations (18)–(19).

(18) $$C_{k}^{cmf}=\left\{\begin{array}{ll} 5\times {\left(S_{k}^{indoor,T}-20\right)}^{2}+0.5 & \;\;\;S_{k}^{indoor,T}<20{}^{\circ}C\\ 0.5\times {\left(S_{k}^{indoor,T}-21\right)}^{2} & \;\;\;20{}^{\circ}C\leq S_{k}^{indoor,T}\leq 22{}^{\circ}C\\ 3\times (S_{k}^{indoor,T}-22{)}^{2}+0.5 & \;\;\;S_{k}^{indoor,T}>22{}^{\circ}C\; \end{array}\right.$$

(19) $$C_{k}^{on/off}=\left|S_{k-1}^{AHU,on/off}-S_{k}^{AHU,on/off}\right|\;\;\;\;\;\;\;s.t.\;\;S^{AHU,on/off}=0,1$$

#### 3.2.3. Optimal Control Method

**Figure 7:**
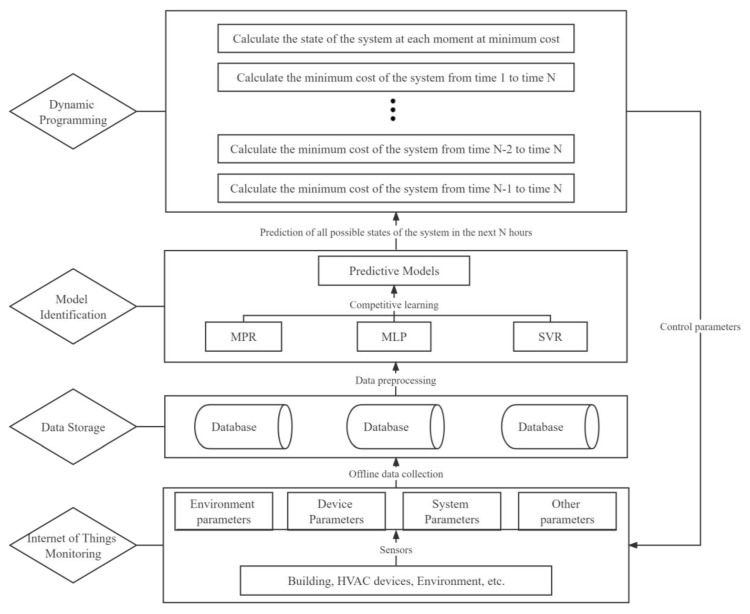
Multistep predictive optimization control method for nearly zero energy residential buildings.

Based on the above considerations, a multistep predictive optimization control method for nearly zero energy residential buildings is proposed, and the control system is simplified as shown in Figure 7.

First, the model identification unit implements three data-driven models (MLP, MPR and SVR) in parallel using historical building operation data, and selects the highest accuracy based on statistical performance metrics. After that, according to the current state of the system and the weather forecast, the state and control paths of the system satisfying the constraint (Equation (16): the room temperature is in the temperature range that may be reached in the previous state, and the remaining start hours of PAU are not greater than the previous state) in the next 24 h are calculated by the time-by-time model. In this case, a directed graph like Figure 6 can be obtained, where the nodes represent the system states and the weights of the links between the nodes represent the cost of the state transfer. Finally, according to the principle of dynamic programming algorithm, the lowest cost is calculated in the reverse direction, the optimal control path is found using this cost in the forward direction and then the time-by-time control parameters for the next 24 h are obtained.

### 3.3. Building Operational Carbon Emissions

From the cradle to the grave, the life cycle of a building is divided into four main phases: construction, operation, demolition and recycling [44]. Energy consumption during the operation phase of the building is the main source of carbon emissions throughout the life cycle and is related to the energy used to operate the building, i.e., the total electricity and natural gas to meet the energy demand. This study reduces the electricity demand during the operation phase of the building by optimizing the control of the HVAC system, thus achieving building emission reduction.

The Intergovernmental Panel on Climate Change (IPCC) developed a model for calculating carbon emissions from the operational phase of buildings, which combines activity-level data and emission factors [45]:

(20) CE=BFS×CEI

(21) $$CEI=\sum_{s}\left(EUI_{s}\times CEF_{s}\right)\;$$

Among them, *CE* is the building carbon emission, in kgCO_2_; *BFS* is the building floor space, in m^2^; *CEI* is the carbon emission intensity, in kgCO_2_/m^2^; *EUI* is the energy use intensity, in kWh/m^2^ for electricity; *CEF* is the carbon emission factor, in kgCO_2_/kWh for electricity; the angular scale s refers to the type of energy. Referring to the Guidance on Greenhouse Gas Accounting and Reporting of China, the carbon emission factor of electricity in this study is 0.5839 kgCO_2_/kWh [46].

## 4. Influence of Thermal Energy Storage in Building

Equation (12) can calculate the minimum cost of the system from state *X*_m−2,i_ to stage *m*; this cost has nothing to do with what state the system is in before stage *m*_−_*2* (the system is transferred from state *X*_m−3,1_ to state *X*_m−2,i_ or state *X*_m−3,n_ to state *X*_m−2,i_), and this step does not need to calculate the optimal path from state *X*_m−2,i_ to stage m (what is *X*_m−2, i_-*X*_m−1,3_-*X*_m,4_ or *X*_m−2,i_-*X*_m−1,1_-*X*_m,2_). This is the requirement of no aftereffect of the dynamic programming algorithm: the state of its previous stages cannot directly affect its future decision making.

The optimal path will only be solved by Equation (13) after the function $\left\{J_{0}^{*},\ldots ,J_{N}^{*}\right\}$ is solved. This makes the algorithm only need to calculate the state transition cost between two adjacent stages in each calculation step, which allows one to solve different types of problems in time O(n^2^) or O(n^3^) for which a naive approach would take exponential time, but at the same time, it increases the difficulty for the algorithm to obtain the historical state of the building. The historical state can provide the algorithm with important additional information that can help its decision making. For example, it can determine if the temperature has been rising in the last few hours and take actions accordingly. Therefore, this paper introduces the variable of thermal energy storage in building. Its definition is shown in Table 4.

The Pearson correlation coefficient is used to reflect the linear correlation between two variables. It can also measure the sensitivity of the nonlinear relationship between the two to a certain extent. Table 7 shows the calculation results of the Pearson correlation coefficients of “thermal energy storage in building” and related parameters. The calculation results show that the “thermal energy storage in building” have a greater impact on the prediction accuracy of the room temperature model and can be used as the historical information input of the room temperature model. It has a small impact on the prediction accuracy of the equipment energy consumption model.

**Figure 8:**
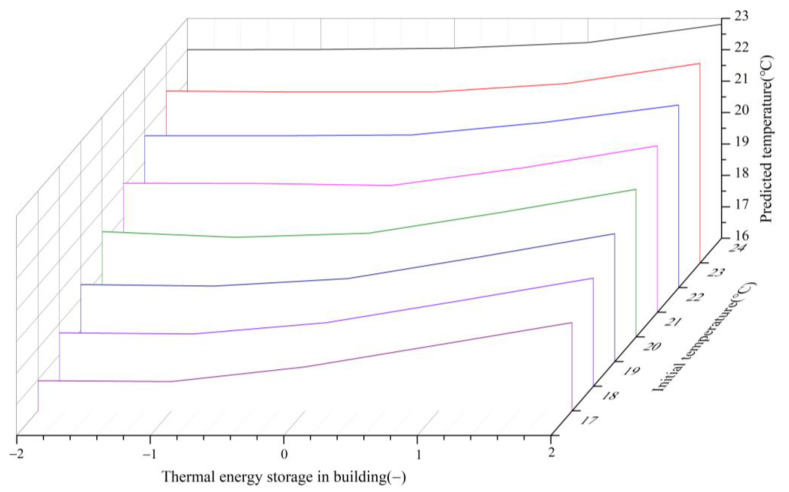
The influence of “thermal energy storage in building” on the predicted value of temperature.

Under the same conditions (sunny day, 12:00, outdoor −3 °C, ASHP runs at maximum power for one hour), the predicted value of the room temperature changes with the “thermal energy storage in building” as shown in Figure 8. In Figure 8, the *X*-axis is the thermal energy storage in building, the *Y*-axis is the initial room temperature and the *Z*-axis is the room temperature after the ASHP runs at maximum power for one hour. It can be seen from the figure that the thermal energy storage in building will affect the room temperature at the next time in the same initial state, and the lower the initial room temperature, the greater the impact.

The influence of ‘thermal energy storage in building’ on the dynamic programming algorithm is directly reflected in the following two aspects: (1) the temperature at which the room reaches a new steady state when the ambient temperature changes and (2) the time step required for the room to reach a new steady state when the ambient temperature changes.

In addition, when the solar radiation is greater than 250 W/m^2^, the “thermal energy storage in building” level of the room should be increased by one level due to the heat gain from the solar radiation.

## 5. Comparison Baseline

A rule-based control strategy is applied as the baseline case to verify the effectiveness of the DP-based HVAC control strategy. The rules used by baseline controller are as follows:

(1) Shading Control: Zhang et al. proposed three shading control strategies based on China’s meteorological conditions [47]. When the solar radiation intensity (southward radiation) received by the facade is higher than 140 W/m^2^, the shading starts to take effect and block 50% of the direct solar radiation. When the shading equipment is activated and the solar radiation intensity is lower than 120 W/m^2^, the shading device closes.
(2) HVAC control: In residential buildings, most of the air conditioning equipment is controlled according to constant set temperature, which is used as the baseline of the control strategy. The on/off control is set according to the personnel presence rate described in Chapter 2.2 (Figure 2). For the sake of comparison, three set temperatures of 18 °C, 20 °C and 22 °C are used in winter. Taking the case that uses 22 °C as the set point as an example, the specific settings are as follows: In winter, when the room temperature is lower than 21 °C and occupation exists, the ASHP starts to operate in heating mode. When the room temperature reaches 23 °C, the ASHP is turned off; that is, the room temperature is controlled at 22 °C ± 1 °C. In summer, when the room temperature is higher than 27 °C and occupation exists, the ASHP cooling mode is activated. When the room temperature drops below 25 °C, the ASHP is turned off; that is, the room temperature is controlled at 26 ± 1 °C.
(3) PAU Control: Considering the influence of latent heat, the PAU is controlled according to the enthalpy difference between indoor and outdoor air. A trade-off between the energy consumption of the PAU and the amount of heat recovery decides the on/off strategy of the PAU. When the outdoor temperature is 22–26 °C, the fan is off and the natural ventilation mode is chosen. The number of the air exchange rate is considered to be 5 times/h. When the outdoor temperature is lower than 22 °C or higher than 26 °C, the PAU is turned on, and its heat recovery device is controlled according to the following rules: (1) Winter: when the indoor air enthalpy is greater than the outdoor air enthalpy by 13 kj/kg, the PAU operates at the heat recovery mode. (2) Summer: when the outdoor air enthalpy is greater than the indoor air enthalpy by 2 kj/kg, the PAU operates at the heat recovery mode. (3) Transition season: the PAU operates continuously in bypass mode.

**Figure 9:**
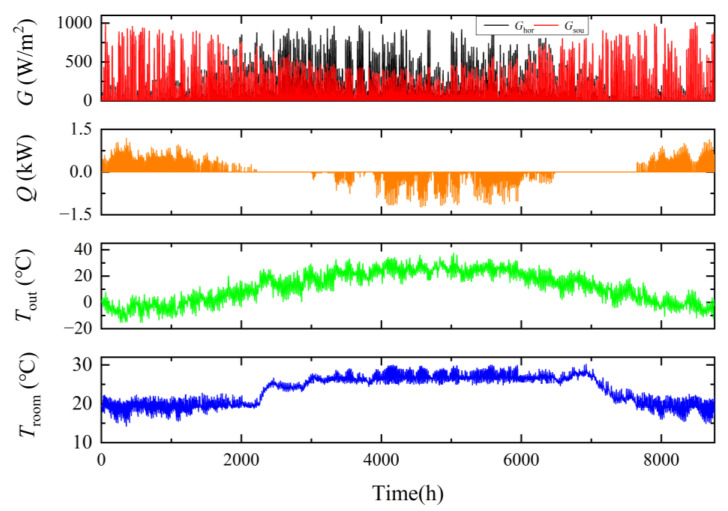
Graph of 8760 h of baseline simulation results throughout the year.

Figure 9 shows the annual simulation results of the baseline simulation when the set temperature is 22 °C in winter and 26 °C in summer. From top to bottom are solar irradiance, heating/cooling effect, outdoor temperature and indoor temperature.

## 6. Results and Discussion

**Figure 10:**
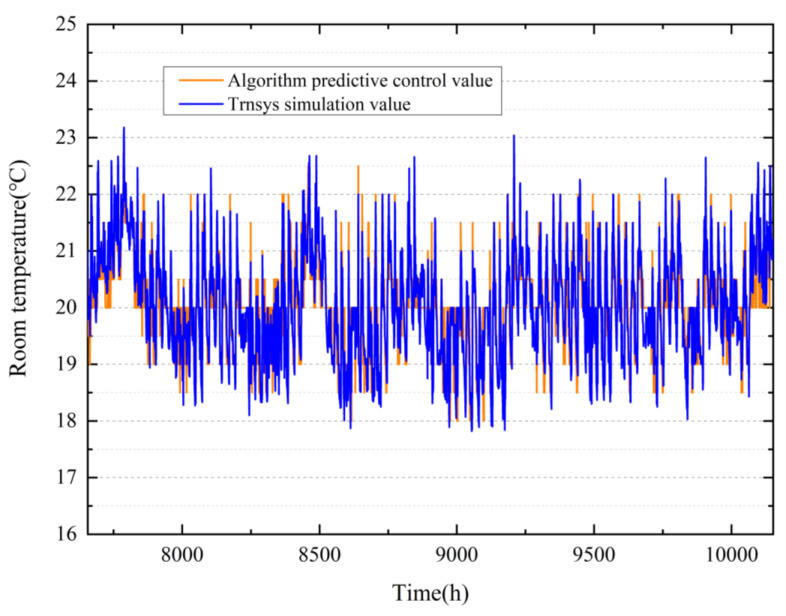
Simulation results of dynamic programming for optimal control of HVAC systems in one heating season. The X-axis is the hour of the year (from 16 November to 28 February of the following year, 7656–10,152).

The research is carried out for the winter condition designated building. The proposed control method (the discomfort cost weighting is 1, and the energy cost weighting is 130) was used to optimize the master bedroom for a heating season (from 16 November to 28 February of the following year, 2497 h) for verification. Figure 10 shows the temperature value set by the dynamic programming algorithm and the simulated temperature from TRNSYS simulation program. Using the proposed algorithm, the energy consumption of the heating season is 96.47 kW·h, and the overall cost is 23,465. The baseline energy consume is 183.37 kW·h, and the overall cost is 26,515. The overall cost is calculated according to Equations (18) and (19), including energy consumption and discomfort penalty item. The algorithm sacrifices a small amount of comfort (causes the room to be 1–2 °C below the comfortable temperature 37% of the time), but it can reduce energy consumption by 47.49%.

**Figure 11:**
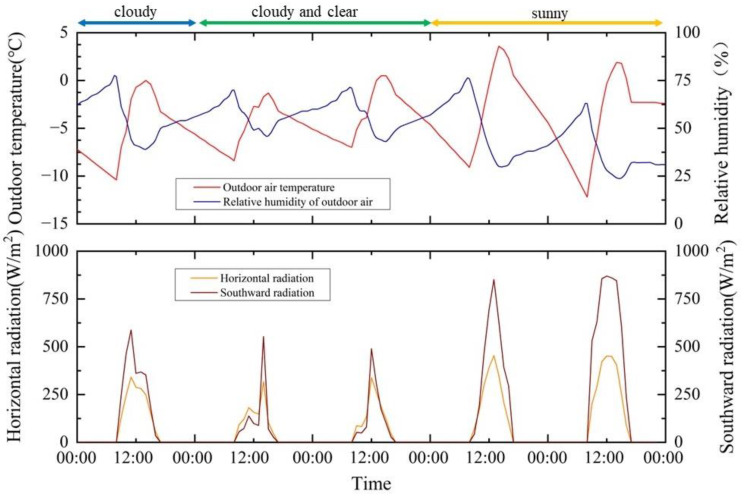
Five consecutive days selected from historical weather data in Beijing.

To further observe the performance of the algorithm, this simulation’s results in the last five days of December are discussed in detail. Figure 11 shows the specific weather data for these five days. The weather conditions in the first three days were more complicated. The first day was cloudy, the second and third days were cloudy and clear and the next two days were sunny with excellent weather conditions.

The initial temperature and relative humidity of the room were set to 20 °C and 30%, respectively. The minimum number of opening hours of fresh air in a room during an optimization period was set to 12 h, which means the fresh air in the room should be turned on for at least 12 h every day. The minimum ventilation hour guarantees enough ventilation to maintain healthy and comfort IAQ environment. Table 8 shows the optimal control output results of the DP algorithm on the first day.

### 6.1. Influence of the Weighting Coefficient

By adjusting the weight coefficient of the discomfort cost and energy cost in the cost function (Equation 17), the user’s lifestyle preference can be adjusted. Increasing the discomfort cost weight or decreasing the energy cost weight can make the controller more focused on thermal comfort and decreasing the discomfort cost weight or increasing the energy cost weight, which can make the controller more focused on energy saving. To make the discomfort cost and energy cost have the same order of magnitude, the discomfort cost weight is set in the range of 1–10, and the energy cost weight is set in the range of 50–150. The objective function of the controller in finding the optimal control path is the lowest overall cost of discomfort cost and energy cost, so the user can find the optimal balance between comfort and energy consumption at any lifestyle preferences (weighting coefficient). Furthermore, the group that makes the lowest overall cost among all combinations of weighting coefficients allows for the controller to find the optimal balance between comfort, lifestyle preferences and energy cost.

**Figure 12:**
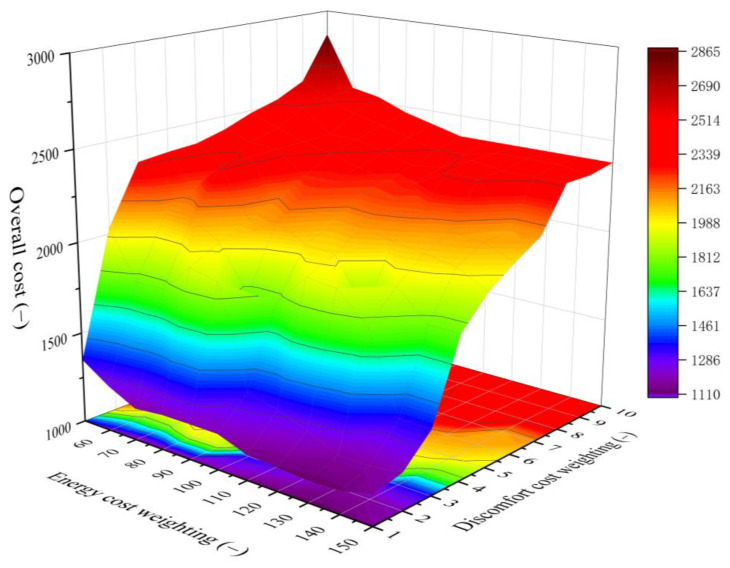
Influence of the weighting coefficient on the overall cost.

Figure 12 shows the influence of the weighting coefficient on the overall cost, using the simulation results from 26 November to 30 November. The overall cost increases monotonically with the increase of the discomfort cost weight, and first decreases and then increases slightly with the increase of the energy cost weight. When the discomfort cost weight is 1 and the energy cost weight is 150, the system obtains the maximum energy saving with the energy consumption of 4.2 kW·h, discomfort cost of 741.36 and overall cost of 1161.36. When the discomfort cost weight is 3 and the energy cost weight is 60, the system obtains the best thermal comfort with the energy consumption of 15.1 kW·h, discomfort cost of 486.89 and overall cost of 1996.89. When the discomfort cost weight is 1 and the energy cost weight is 130, the system achieves the best optimization with the energy consumption of 5.72 kW·h, discomfort cost of 542.54 and the overall cost of 1114.54. In addition, if the weight coefficients are set unreasonably (e.g., the discomfort cost weight is set too high), the controller based on the dynamic programming does not obtain better optimization results than the baseline.

### 6.2. Performance of Thermal Comfort

**Figure 13:**
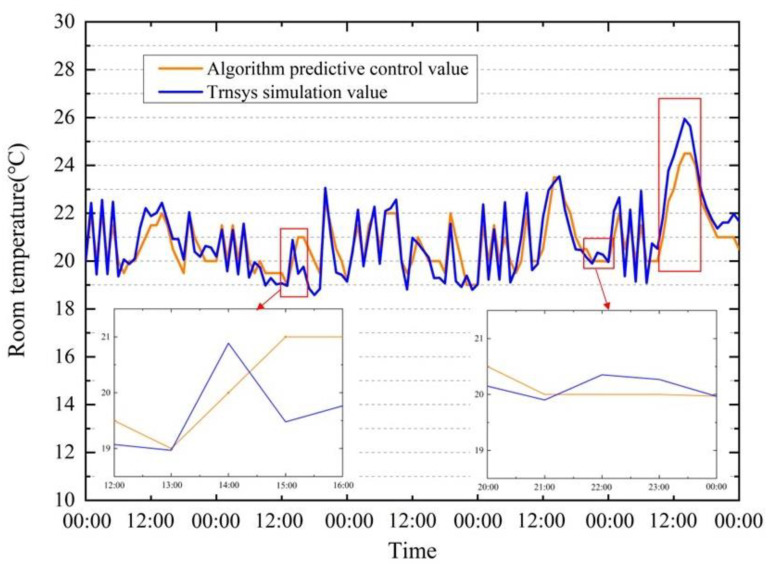
Simulation results of room temperature.

Figure 13 shows the predicted temperature from the ANN model and the simulated temperature from TRNSYS simulation program over five days. The results show that the proposed algorithm can effectively use the thermal inertia of the building and select the appropriate time to preheat the building according to the outdoor weather. For example, the algorithm will intelligently choose an opportunity to increase the room temperature between 17:00 and 20:00 to make full use of heat intake by solar radiation and reduce the duration of the use of ASHP at night. For another example, in the second or third day of cloudy to fine weather, the algorithm turns on the ASHP intermittently on cloudy days and sets a suitable temperature to maintain the heat in the room, so that the ASHP avoids working when the sky is clear.

The identified room temperature model is the basis of the proposed method. In some cases, the accuracy of the model needs to be improved: (1) The time step of the control scheme should be flexibly set according to the case requirement. The time step used in this research is one hour, which is the most commonly used period in practice. For the cases where the weather condition changes rapidly and constantly, a shorter time step may be appropriate. The inaccuracy of the room temperature prediction in Figure 13a is due to outdoor weather fluctuations. At the 38th hour, the southward solar radiation value is 553.55 W/m^2^, so the temperature model predicts that the room temperature will continue to rise, while the actual weather quickly turns cloudy, and the room temperature drops. (2) The temperature model cannot correctly reflect the influence of the internal heat source on the indoor temperature precisely. The slight rise in room temperature in Figure 13b is due to the electrical appliances and lights in the room being turned on, and the room temperature model does not reflect this change. (3) Limited by the data set used for model training, when the southward radiation is greater than 600 W/m^2^, the predicted indoor temperature rise rate is slightly lower than the actual value, as shown in Figure 13c.

The comfortable temperature range of the room is set at 20–22 °C. During the five days of optimal control using dynamic programming, the room was at an uncomfortable temperature 47.0% of the time. Among them, the room temperature was 19–20 °C 20.8% of the time, the room temperature was 18–20 °C 9.1% of the time and the room temperature more than 22 °C 16.6% of the time. The low temperature mostly occurs in the early morning, and the high temperature mostly occurs at noon. Using the dynamic programming algorithm for control, the calculation result of discomfort penalty item is 542.54, and baseline-20 °C is 363.75.

### 6.3. Performance of Energy Saving and Emission Reduction

**Figure 14:**
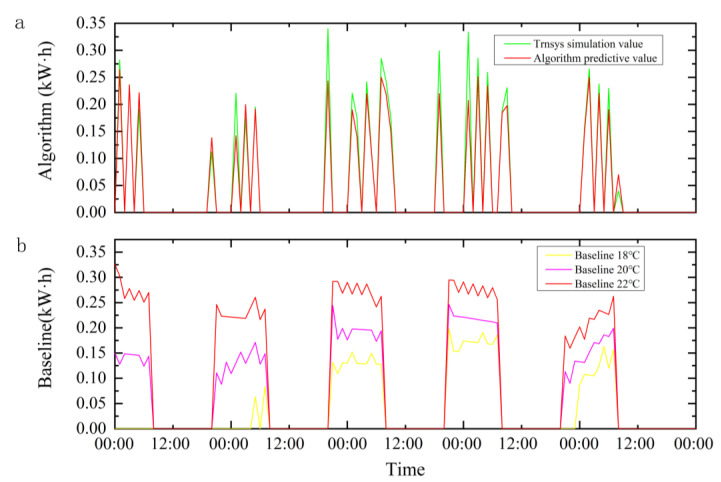
ASHP energy consumption.

Based on the above results, our data are meaningful for further research on the energy saving and emission reduction potential of dynamic programming algorithms. Figure 14 shows the results of the energy consumption. Figure 14a gives the energy consumption when using the proposed method and the predicted energy consumption of the identified energy model. Figure 14b gives the energy consumption when using baseline control strategy of fixed set temperature of 18 °C, 20 °C and 22 °C. At the set temperature of 18 °C, there is no energy consumption on the first day because the initial room temperature is set to 20 °C.

In Figure 14a, the red line is the predicted energy consumption value calculated by the energy consumption model, and the green line is the TRNSYS simulation value. Since dynamic programming has no aftereffect requirements, the algorithm cannot obtain the historical on/off status of the ASHP. This will cause the predicted energy consumption of the ASHP to be lower than the TRNSYS simulation value when it is turned on after a long-term shutdown. Setting the continuous on/off time of the ASHP as the state information of the dynamic programming algorithm may solve this problem. However, as mentioned earlier, when the dimensionality of the state variables increases, the computational complexity of dynamic programming problems grows exponentially.

Compared to the baseline-20 °C, the dynamic programming algorithm has 35.1% energy saving and emission reduction. The reason is that, during periods of high outdoor temperature, the baseline control strategy keeps the setpoints at 20 °C, which leaves the ASHP in ‘on’ status for an unnecessarily long time and brings excessive energy consumption. By comparison, dynamic programming algorithms coordinate the temperature setting with weather factors and building thermal inertia, resulting in a more economical control strategy. For example, the algorithm chooses to turn on the ASHP for a short time in the early morning (00:00–09:00) according to the weather of the day and the heat storage state of the building to provide heat to the controlled building; it turns off the ASHP during the day and evening (09:00–20:00) with suitable weather conditions to make full use of the free natural heat; at night (20:00–24:00), the ASHP is turned on at an appropriate time to maintain the thermal comfort of the building. Compared with the traditional rule-based control strategy, the proposed algorithm actively responds to the outdoor environment change and reduces the energy consumption without sacrificing the indoor thermal comfort.

### 6.4. Trade-Off between Thermal Comfort and Energy Saving

There is a trade-off between thermal comfort and energy consumption, and the dynamic programming algorithm trades off some thermal comfort for energy savings. The results in Table 9 gives the energy cost, discomfort cost, uncomfortable hours and overall cost. Energy cost is the energy consumption value multiplied by 100; the discomfort cost is calculated by Equation 18, which is a quadratic function. Its discomfort cost increases with departure from the comfort zone (20–22 °C) and has a higher cost at low temperatures. Uncomfortable hours are times when the room temperature is outside the comfort zone. The overall cost is the sum of the energy cost and the discomfort cost.

The proposed algorithm maintained the indoor thermal environment at the comfort level for the 24 h, while the baseline case only regulates the indoor thermal environment during the occupied period (Figure 2). The controlled building has an envelope structure with high thermal insulation performance and high air-tight performance, reaching the energy efficiency level of nearly zero energy consumption buildings. Under the baseline-22 °C control strategy, the room will be overheated at noon and the temperature will be higher than 22 °C. Therefore, the discomfort cost of baseline-22 °C is higher than that of the dynamic programming algorithm. Compared with baseline-22 °C, the dynamic programming algorithm prevents overheating through intelligent control, which reduces the cost of room discomfort by 18.7% and saves energy by 56.3%. The dynamic programming algorithm reduces the overall cost by 43.6% compared with baseline-22 °C; compared with baseline-20 °C, the discomfort cost of dynamic programming algorithm is increased by 49.1%, but both are at a lower level. The dynamic programming algorithm’s energy cost was reduced by 35.1%, and the overall cost was reduced by 10.5%; compared with baseline-18 °C, the energy cost is only increased by 27.9%, but its discomfort cost is reduced by 70.1%, and the overall cost is reduced by 51.8%.

The above analysis shows that the dynamic programming algorithm balances the relationship between indoor thermal comfort and energy consumption by allowing for the room to be temporarily in an uncomfortable state in exchange for energy saving potential. The dynamic programming algorithm can find the control strategy with the lowest overall cost.

### 6.5. Future Directions

The future work is mainly concentrated in the following aspects:

(1) The identification of an accurate model requires a big historical data set, which is difficult to obtain in some buildings. Further research should address how to generate an acceptable model with a smaller data set.
(2) The evaluation of thermal comfort in this paper is based on room temperature and does not consider the influence of other parameters such as humidity and mean radiant temperature. There are two reasons for this: on the one hand, parameters related to thermal comfort need to be predicted using the model, and introducing more parameters increases the complexity of the model, and on the other hand, the computational complexity of the dynamic programming problem grows exponentially with the number of dimensions of the state variables. Further research is needed to perform a more objective thermal comfort evaluation without significantly increasing the computational cost.
(3) It is important to deploy the developed algorithm into a real-world testbed. From the simulation in the real world, which is extremely complex, there are some challenges related to the infrastructure required to effectively deploy the algorithm. Future work will be focused on these aspects, as well as the performance assessment of the DP control strategy on site deployment.

## 7. Conclusions

This research proposes a heating control method for HVAC based on dynamic programming to achieve better balance between thermal comfort, energy consumption and personnel preferences of a nearly zero energy residential building. Firstly, the simulation program for a nearly zero energy residential building is established in the TRNSYS environment. Then, three model identification methods, namely multivariate linear regression model, support vector regression model and Artificial Neural Network, were used to establish room temperature and HVAC energy consumption models. The suitable models are chosen according to the statistical performance criteria among the three model identification methods. Finally, based on the selected models, the dynamic programming algorithm is used for the model predictive control of the system. The main conclusions of this research are as follows:

(1) Compared with the simplified first principle models used in the existing literature, the room temperature model established by the data-driven technology in this research reflects the characteristics of the building with better precision.
(2) Using the average room temperature 4 h before each optimal control to characterize the thermal energy storage in building can solve the problem that the dynamic programming has difficulty obtaining the building historical state due to no aftereffect requirement, so that the building model used by the controller can better reflect the building thermal inertia.
(3) By adjusting the weighting coefficients of discomfort cost and energy cost in the cost function, the lifestyle preferences of users can be adjusted. When the discomfort cost weight is 1 and the energy cost weight is 130, the overall cost is the lowest, i.e., the optimal balance between end-user comfort, lifestyle preferences and HVAC system energy consumption is achieved.
(4) The Python/TRNSYS co-simulation results show that the proposed control scheme can adjust the HVAC system and the operation mode the building not only according to the current monitored information, but also including consideration of future environment change and predicted response of the building thermal system. Its overall cost is far lower than the case using rule-based control. Compared with the baseline-20 °C case, which keeps the room temperature at the lowest comfort level, the proposed control algorithm can save energy and reduce emissions by 35.1%.

## Figures and Tables

**Table 1:** Main input parameters of the model.

Parameters	Values	
Envelope	Residential Unit	Experimental Unit
External wall heat transfer coefficient (W/(m^2^· K))	0.2	0.15
External wall solar radiation absorption coefficient (−)	0.7	0.7
Inner wall solar radiation absorption coefficient (−)	0.4	0.4
Heat transfer coefficient of outer window (W/(m^2^· K))	0.8	0.8
Shading coefficient of exterior window (−)	0.5	0.5
Outer door heat transfer coefficient (W/(m^2^· K))	0.8–1.0	0.8–1.0
Cold wind penetration (1/h)	0.1	0.1
**Primary Air Unit**		
Fresh air volume (m^3^/h)	150	
Power (W)	45	
Heat recovery efficiency (enthalpy efficiency)	75%	
**Air-Sourced Heat Pump**	**bedroom**	**living room**
Rated cooling capacity (Kw)	2.36	2.6
Cooling power (Kw)	0.717	0.8
Rated heating capacity (Kw)	2.65	2.86
Heating power (Kw)	0.683	0.81

**Table 2:** Internal heat source parameters.

Room	Lighting Power Density Value (W/m^2^)	Equipment Power (W)	Human Body Heat Dissipation (W/Person)
bedroom	5	50	60
living room	5	150	75
bathroom	5	50	75
kitchen	5	250	75

**Table 3:** Description of simulation conditions.

Day		Night		Shading Control	Temperature Setting (°C)
ASHP On/Off	PAU On/Off	ASHP On/Off	PAU On/Off		
0	0	0	0	0/0.5	−
0	0	1	0	0/0.5	18/20/22/24
0	1	0	1	0/0.5	18/20/22/24
0	1	1	1	0/0.5	18/20/22/24
1	0	0	0	0/0.5	18/20/22/24
1	0	1	0	0/0.5	18/20/22/24
1	1	0	1	0/0.5	18/20/22/24
1	1	1	1	0/0.5	18/20/22/24
random	0	random	0	0/0.5	18/20/22/24
random	1	random	1	0/0.5	18/20/22/24

Note: 0 means off, 1 means on, 0.5 means blocking 50% of solar radiation.

**Table 4:** Thermal energy storage in building.

Room Temperature	Warm and Cold Level	Variable
T ≤ 17 °C	Very cold	−2
17 °C ≤ T ≤ 19 °C	Cold	−1
19 °C ≤ T ≤ 1 °C	Medium	0
21 °C ≤ T ≤ 23 °C	Warm	+1
T ≥ 23 °C	Very warm	+2

**Table 5:** Data-driven model input.

Room Temperature Prediction Model										
time	$T_{k}^{his}$	$S_{k}^{out,T}$	$S_{k}^{out,Rh}$	$S_{k}^{G,hor}$	$S_{k}^{MAU,\mathrm{mod}e}$	$S_{k}^{MAU,speed}$	$S_{k}^{in,T}$	$Q_{k}^{}$		
**ASHP energy consumption model**										
time	$T_{k}^{his}$	$S_{k}^{out,T}$	$S_{k}^{out,Rh}$	$S_{k}^{G,hor}$	$S_{k}^{MAU,\mathrm{mod}e}$	$S_{k}^{MAU,speed}$	$S_{k}^{in,T}$	$S_{k}^{AHU,\mathrm{mod}e}$	$S_{k+1}^{in,T}$	$D_{k,k+1}^{in,T}$

Note: *Q* means ASHP cooling capacity; *D* means ASHP energy consumption.

**Table 6:** The selected models and their accuracy.

Model Name		Model Structure	*R* ^2^	*MAE*	*MSE*	*MAPE*
Temperature	MLR	Highest term: 5	0.9231	0.0285	0.0014	8.66%
	SVR	Kernel function: Gaussian	0.9154	0.0315	0.0015	9.53%
	ANN	Relu(150,50)	0.9574	0.0513	0.0023	5.52%
Consumption	MLR	Highest term: 4	0.8643	0.0323	0.0024	9.73%
	SVR	Kernel function: Gaussian	0.9126	0.0299	0.0015	8.97%
	ANN	Relu(150,250)	0.9548	0.0136	0.0007	7.65%

Note: Relu(150,50) indicates that the ANN activation function is Relu, with two hidden layers: 150 nodes in the first layer, and 50 nodes in the second layer.

**Table 7:** Pearson correlation coefficient calculation results between “thermal energy storage in building” and related variables.

Related Variables	“Thermal Energy Storage in Building” and Room Temperature	“Thermal Energy Storage in Building” and ASHP Energy Consumption
Pearson correlation coefficients	0.77	0.04

**Table 8:** The output value of the first day of the dynamic programming algorithm.

Time	Temperature Setting/Predicted Value	PAU Minimum Open Hours Remaining	ASHP On/Off	PAU On/Off	Shade State
0	20.00	11	1	1	0
1	22.00	10	0	1	0
2	20.00	10	1	0	0
3	22.00	9	0	1	0
4	20.00	9	1	0	0
5	21.50	8	0	1	0
6	20.00	8	0	0	0
7	19.50	8	1	0	0
8	20.00	7	1	1	0
9	21.00	7	0	0	0
10	20.00	6	0	1	0
11	20.50	5	0	1	0
12	21.50	4	0	1	0
13	21.50	3	0	1	0
14	21.50	3	0	0	0
15	21.50	2	0	1	0
16	21.00	2	0	0	0
17	20.50	1	0	1	0
18	20.00	1	0	0	0
19	19.50	0	1	1	0
20	21.00	0	0	0	0
21	20.50	0	0	0	0
22	20.00	0	0	0	0
23	19.00	0	0	0	0

**Table 9:** Results for the cost function.

Control Strategy	Energy Cost (−)	Discomfort Cost (−)	Uncomfortable Hours (−)	Overall Cost (−)	Carbon Emissions (kgCO_2_)
DP algorithm	572.00	542.54	56	1114.54	3.34
Baseline-18 °C	447.00	1867.43	120	2314.43	2.61
Baseline-20 °C	881.00	363.75	15	1244.75	5.14
Baseline-22 °C	1309.00	667.25	22	1976.25	7.64

## Data Availability

Not applicable.

## Nomenclature

$T_{k}^{his}$	thermal energy storage in building (−)
$x_{k}^{}$	the state of the system (−)
$u_{k}^{}$	decision variable (−)
$J_{k}^{*}$	the lowest cost from stage *k* to stage *m* (−)
$S_{k}^{in,T}$	current temperature of the room (°C)
$S_{k}^{PAU,times}$	the remaining start hours of the PAU (−)
$S_{k}^{ASHP,on/off}$	the on/off of the ASHP (−)
$S_{k}^{Set,T}$	the setting of the room temperature (°C)
$S_{k}^{PAU,on/off}$	the on/off of the PAU (−)
$S_{k}^{PAU,mode}$	the working mode of the PAU (−)
$S_{k}^{shading}$	solar radiation blocked by shading equipment (%)
$S_{k,\min}^{in,T}$	minimum room temperature at time k (°C)
$S_{k,\max}^{in,T}$	maximum room temperature at time k (°C)
$S_{k}^{Weather}$	outdoor weather (−)
$\lambda_{k}^{cmf}$	the weighting coefficient of discomfort cost (−)
$\lambda_{k}^{cst}$	the weighting coefficient of energy cost (−)
$\lambda_{k}^{on/off}$	the weighting coefficient of ASHP on/off cost (−)
$C_{k}^{cmf}$	discomfort cost (−)
$C_{k}^{cst}$	energy cost (kW)
$C_{k}^{on/off}$	ASHP on/off cost (−)
G	solar radiation (W/m^2^)
Q	the heating and cooling output of ASHP (kW)
Subscripts	
0−24,k	the stage index
Abbreviations	
HVAC	heating, ventilation and air conditioning
NZE	net zero emissions
NZEB	nearly zero energy buildings
MPC	model predictive control
SVM	support vector machine
ANN	Artificial Neural Network
MPR	multivariate polynomial regression
*MSE*	Mean Square Error
*MAE*	Mean Absolute Error
*MAPE*	Mean Absolute Percent Error
*R* ^2^	coefficient of determination
RBFs	radial basis functions
MLP	multi-layer-perceptons
ASHP	Air-Sourced Heat Pump
PAU	Primary Air Unit
BNMI	best network after multiple iterations
DP	dynamic programming
